# Supplementary material for: Clinical Characteristics of Atrial Flutter and Its Response to Pharmacological Cardioversion with Amiodarone in Comparison to Atrial Fibrillation
Source: J Clin Med. 2023 Jun 25;12(13):4262. doi: 10.3390/jcm12134262 (PMC10342954; doi:10.3390/jcm12134262)
Supplement: Supplementary file 1 [file jcm-12-04262-s001.zip › jcm-2449460-supplementary.pdf]

Supplementary Table S1 Different demographic and clinical variables in the cohort of patients with atrial flutter depending on the successful termination of arrhythmia

**Variable**	**Return of sinus rhythm N=180**	**Persistence of arrhythmia upon discharge N=42**	**P-value**
Male sex	116 (64.4%)	29 (69.1%)	0.573
Age, years	67.1 ± 11.9	68.3 ± 10.3	0.875
Weight, kg	86.7 ± 21.5	84.2 ± 18.2	0.713
Hospitalization time, days	3.3 ± 2.4	2.8 ± 2.4	0.058
Duration of AFl episode, hours	48 (15; 168)	72 (49; 120)	0.755
Duration of episode >48h	73 (65.8%)	22 (75.9%)	0.300
Persistent AFl	61 (51.7%)	13 (44.8%)	0.508
Heart rate, bpm	113.4±37.2	123.1 ± 43.7	0.309
Heart rate >130 bpm	60 (35.3%)	15 (41.7%)	0.470
EHRA class	2 (2; 3)	3 (1; 3)	0.324
CHA_2DS_2-VASc, pts	3 (2; 4)	3 (1; 5)	0.714
Arterial hypertension	123 (73.7%)	28 (77.8%)	0.607
Diabetes mellitus	48 (28.9%)	15 (41.7%)	0.134
CAD/PAD	54 (32.1%)	14 (37.8%)	0.505
Former TIA/stroke	18 (10.8%)	3 (8.3%)	0.662
History of PVI	19 (11.2%)	1 (2.8%)	0.120
LVEF, %	47.8 ± 12.4	44.4 ± 11.9	0.042
LVEF <50%	54 (35.3%)	18 (54.6%)	0.039
LAd, mm	43.2 ± 6	44.6 ± 6.3	0.497
LAd >40 mm	114 (77.6%)	25 (78.1%)	0.944

TnT >0.014 pg/mL	44 (49.4%)	19 (70.4%)	0.056
SCr, mg/dL	1.2±0.9	1.1±0.7	0.493
eGFR, mL/min	67.7 ± 18.4	69.4 ± 19.1	0.542
eGFR <60 mL/min per 1.73 m2	52 (31.7%)	10 (26.3%)	0.516
Potassium level, mEq/L	4.4 ± 0.4	4.3 ± 0.5	0.136
WBC ×1000/μL	8.1 ± 3	8.3 ± 2.3	0.206
Hemoglobin, g/dL	14 ± 2	14.1 ± 1.5	0.995
TSH, mIU/L	2.8 ± 5.6	1.6 ± 1.1	0.101

AFl- atrial flutter; AF – atrial fibrillation; bpm- beats per minute; eGFR – estimated glomerular filtration rate; EHRA – European Heart Rhythm Association; CAD – coronary artery disease; LAd- left atrial diameter; LVEF – left ventricular ejection fraction; PAD – peripheral artery disease; TIA – transient ischemic attack; PVI – pulmonary vein isolation; TnT- troponin T; SCr – serum creatinine concentration; WBC – white blood cell count; TSH – thyroid-stimulating hormone
